# Cancer Development Associated with Cardiovascular Diseases

**DOI:** 10.31662/jmaj.2022-0166

**Published:** 2022-10-06

**Authors:** Tomohiro Fujisaki, Kenshi Yamanaga, Eiichiro Yamamoto, Kenichi Tsujita

**Affiliations:** 1 Department of Cardiovascular Medicine, Graduate School of Medical Sciences, Kumamoto University, Kumamoto, Japan

**Keywords:** Cardio-oncology, Coronary artery disease, Cancer, Polyvascular disease

Cardiovascular disease and cancer are two distinct disease entities; these might intersect with each other and share common pathophysiology and mutual risk factors ^(1)^. The concurrence of cancer and cardiovascular disease has been increasing owing to the aging population with more cancer survivors and cardiovascular disease survivors due to improving treatment strategies, leading to a new discipline foundation called “Cardio-oncology”. The relationship between the two appears to be bidirectional, with a growing body of literature demonstrating an increased risk of cardiovascular disease among patients with cancer and vice versa ^(1), (2), (3)^. These links between existing cancer and subsequent cardiovascular disease are much appreciated since cardiologists often care for cancer survivors with cardiotoxicity secondary to emerging targeted oncology therapies. In contrast, it is less appreciated that individuals with cardiovascular disease have a higher subsequent cancer risk, highlighting the need for further robust studies to understand the intertwined relationship between cardiovascular diseases and subsequent cancer.

In this issue of the *JMA Journal*, Suzuki et al. performed a retrospective analysis of patients with coronary artery disease identified from a Japanese single-center registry ^(4)^. They stratified patients with coronary artery disease according to the presence of polyvascular disease (PVD). In this registry, PVD was defined as an aortic disease, peripheral arterial disease, or other arterial diseases at enrollment. The study did not include cerebrovascular disease as a category of PVD. They compared the incidence of cancer and mortality risk of the two groups. After excluding 236 patients, the study included 8,856 patients, with 74% of them being male, and the median age was 70 years old. Among the total patients, only 716 (8.1%) patients were classified into the PVD group. There were significant differences in patient demographics between the two groups, which could have led to considerable bias and affected the study outcomes. For example, there were more elderly and male patients; a higher prevalence of hypertension, chronic kidney disease, previous stroke, cigarette use, previous myocardial infarction, and heart failure was observed in the PVD group. Furthermore, more patients underwent coronary artery bypass grafting in the PVD group. Suzuki et al. tried to account for this using propensity score matching to minimize the bias of systematic differences between the two groups and showed excellent covariate balance post-matching.

Suzuki et al. showed a higher cancer incidence in this cohort with PVD. Cox proportional hazards regression models confirmed that the presence of PVD was associated with the incidence of cancer (adjusted hazard ratio (HR): 1.36; 95% confidence interval (CI): 1.03-1.77). All-cause mortality (15.2% vs. 5.4%, P < 0.0001), cardiovascular death (5.2% vs. 2%, P < 0.0001), and cancer mortality (2.5% vs. 1.3%, P = 0.0073) were higher in the PVD cohort compared with the other cohort. Landmark sensitivity analyses further revealed that the more the coexisting vascular diseases, the higher the cancer incidence. These findings suggest a potential association between the severity of atherosclerotic diseases and cancer development. Suzuki et al. should be commended for these thoughtfully conducted analyses that complement previous studies with a limited understanding of the association between the extent of PVD and subsequent cancer risk ^(5)^. The strengths of this study are the large-scale study size, robust statistical analysis methods, prospective ascertainment of vascular disease and cancer, and long-term follow-up periods. However, there were some limitations to this study. For example, the nature of single-center observational analysis was subject to residual confounding, and causal relationships could not be drawn. The study revealed an association between the severity of atherosclerotic diseases and cancer development, however, it might just represent a shared consequence of an underlying process or it may be due to confounding by currently unknown variables.

How can the current work by Suzuki et al. implicate our practice? The study alarmed clinicians to be vigilant of the increased risk of subsequent cancer in patients with cardiovascular disease, particularly in patients with PVD. The message is important because it is currently unknown which subgroup of patients with cardiovascular disease may benefit from early or extended cancer screening. Further studies are warranted to investigate the complicated relationship between the two disease domains to enable timely cancer detection and treatment in patients with cardiovascular disease.

## Article Information

### Conflicts of Interest

None
